# The Economic Costs of Progressive Supranuclear Palsy and Multiple System Atrophy in France, Germany and the United Kingdom

**DOI:** 10.1371/journal.pone.0024369

**Published:** 2011-09-08

**Authors:** Paul McCrone, Christine Anne Mary Payan, Martin Knapp, Albert Ludolph, Yves Agid, P. Nigel Leigh, Gilbert Bensimon, for the NNIPPS Study Group

**Affiliations:** 1 Health Service and Population Research Department, Institute of Psychiatry, King's College London, London, United Kingdom; 2 APHP, Département de Pharmacologie Clinique, Hôpital de la Pitié-Salpêtrière, UPMC Pharmacologie, Paris 6, UMR 7211, Paris, France; 3 Personal Social Services Research Unit, London School of Economics, London, United Kingdom; 4 Department of Neurology, University of Ulm, Ulm, Germany; 5 ICM (Brain and Spine Institute), Hôpital de la Pitié-Salpêtrière, Assistance Publique Hôpitaux de Paris & UPMC Université Paris 06, Paris, France; 6 Brighton and Sussex Medical School, Trafford Centre for Biomedical Research, University of Sussex, Falmer, Brighton, United Kingdom; University of South Florida College of Medicine, United States of America

## Abstract

Progressive supranuclear palsy (PSP) and multiple system atrophy (MSA) are progressive disabling neurological conditions usually fatal within 10 years of onset. Little is known about the economic costs of these conditions. This paper reports service use and costs from France, Germany and the UK and identifies patient characteristics that are associated with cost. 767 patients were recruited, and 760 included in the study, from 44 centres as part of the NNIPPS trial. Service use during the previous six months was measured at entry to the study and costs calculated. Mean six-month costs were calculated for 742 patients. Data on patient sociodemographic and clinical characteristics were recorded and used in regression models to identify predictors of service costs and unpaid care costs (i.e., care from family and friends). The mean six-month service costs of PSP were €24,491 in France, €30,643 in Germany and €25,655 in the UK. The costs for MSA were €28,924, €25,645 and €19,103 respectively. Unpaid care accounted for 68–76%. Formal and unpaid costs were significantly higher the more severe the illness, as indicated by the Parkinson's Plus Symptom scale. There was a significant inverse relationship between service and unpaid care costs.

## Introduction

Progressive supranuclear palsy (PSP) and multiple system atrophy (MSA) are rare progressive neurodegenerative disorders, usually presenting as akinetic-rigid syndromes (‘parkinson plus syndromes’). Age at onset is typically between 55 and 65, and the average life expectancy from onset is 5–10 years for both diseases [1]–[8]. The prevalence of each of these disorders has been estimated at 2–7 per 100,000 [4], [5], [9]–[15], although this is likely to be an underestimate because of diagnostic confusion with idiopathic Parkinson's disease. The number of people aged 55 or above with PSP has been estimated at 14–25 per 100,000, with a figure of 17–29 per 100,000 for MSA [5], [10]. Given that there are approximately 177 million people aged 55 or over in the 27 countries of the EU [16] this translates to 25,000–44,000 with PSP and 30,000–51,000 with MSA.

Patients with MSA and PSP require care from a range of services, and it is likely that such inputs will need to increase as the disease progresses. However, to date there have been no attempts to comprehensively measure and cost service use for patients. Measures of costs for a representative sample of patients allow us to determine relationships between care inputs and patient needs, assess the cost-effectiveness of specific treatments and highlight the impact that these conditions have on society as a whole.

As part of the NNIPPS study [17] we adapted a health service use questionnaire [18] to estimate the resource use associated with PSP and MSA in three European countries (France, Germany and the UK). This paper reports the service use and cost for patients at entry to the study and identifies predictors of the costs.

## Methods

### Ethics approval

Patients gave written consent prior to participating in the study. The protocol and amendments were reviewed and approved by the Comité de Protection des Personnes of Pitié-Salpêtrière Hospital (France), the UK Multicentre Research Ethics Committee (MREC), (UK), Ethikkommission of the University of Ulm, (Germany), and by local Institutional Review Boards (Ethics Committees) where appropriate (UK, Germany). These include, UK: Ethics committees of Belfast, Birmingham - City Hospital, Birmingham - Queen Elizabeth Hospital, Cambridge, Liverpool, King's College London, NHNN & Queen Square Hospital, Newcastle upon Tyne, Stafford, Aberdeen, Guernsey, Swansea. Germany: Aachen, Berlin, Bochum, Dresden, Freiburg, Halle, Hannover, Magdeburg, München, Regensburg, Rostock, Tübingen, Ulm.

The broad aims of the NNIPPS project were to evaluate the use of riluzole, a potential neuroprotector and to assess the natural history of PSP and MSA. Details of the inclusion criteria and study design are published elsewhere [17]. Briefly, patients with an akinetic-rigid syndrome diagnosed as PSP or MSA according to the NNIPPS diagnostic criteria were eligible. The intention to treat (ITT) population comprised of 760 patients (362 PSP and 398 MSA) recruited in 44 centres in the UK, France and Germany. Patients were stratified according to diagnosis and randomised double-blind to riluzole or placebo. The primary efficacy measure was survival, and secondary endpoints included rate of change in functional scores.

Patients were to be followed-up 3-monthly for three years for safety assessment. For efficacy and natural history assessment, the following measures were completed at entry to the study (between 2000 and 2002) and used in the subsequent economic analyses: (i) demographic characteristics (including age, gender, ethnicity, education), (ii) vital signs (including weight and height), (iii) Hoehn and Yahr disease staging scale (HYS) [19], (iv) a Short Motor Disability Scale (SMDS) [21], (v) Clinical Global Impression of Disease Severity (CGI)-ds [20], (vi) Parkinson Plus Symptoms scale (NNIPPS-PPS) [21], (vii) Short-Form 36 [22], (viii) Client Service Receipt Inventory (CSRI) [18]. SMDS was completed at follow-up 3-montly, HYS, CGI-ds, PPS, 6-monthly, and SF36 and CSRI 6-monthly the first year then 12-monthly.

### Service use and costs

It is important in any economic study to include information on all services that may be used because of the specific condition under investigation. Service use during the six-month period prior to interview was measured using the Client Service Receipt Inventory (CSRI) adapted for these disorders (Appendix S1). Participants were asked for information on their use of hospital and community services, i.e. whether they used them, how many times and (where relevant) for the average duration of contacts. They were also asked to provide details of any scans or tests they had received in the six-month period, whether they had been provided with any prostheses (wheelchairs, walking frames, etc), and whether any adaptations had been made to their home because of their illness. Further information was sought on care provided because of the illness by family members or friends and any impact that the illness had on employment of the patients themselves or members of their families.

Unit costs were attached to the service use measures in order to generate service costs and were obtained from a number of sources. UK unit costs were taken from a recognised national source [23]. Figures for most services in France and Germany were obtained from Medtap International [24]. A small number of costs were not available and therefore they were based on the costs from other countries and adjusted using ratios between the countries for costs that were available. Most figures from the UK represented actual costs whilst those provided by Medtap International represented a mixture of costs and charges. Care is thus required in making comparisons between countries.

The study recorded personal care input from family members and friends. Whilst care from friends or family members is rarely paid for, it still represents an important economic cost as the time spent caring could have been used in alternative activities. We made the assumption that if the family member or friend was not providing care then this would have to be provided by services and consequently we valued unpaid care at the cost of a home care worker. (Similarly, we could have argued that if the family member or friend was not caring for the patient then they could in theory use their skills to provide care in a paid capacity for someone else.).

Data on medications received were recorded for each patient in the study. Medication names were entered as free-text variables and approximately 1000 different names were recorded (some being the same medications with slightly different spellings or brand names). A pragmatic approach was taken whereby only medication taken by at least 1.5% of the sample was costed. The costs of drugs were obtained where possible from the British National Formulary (the most common source for such data in the UK) and combined with medication levels and period of receipt. Medication costs were subsequently converted to Euros by dividing by 0.663 (the exchange rate at the time of the study) [25]. All costs were inflated to 2007/8 prices and UK costs converted to Euros. The unit costs of specific services used in the study are shown in Appendix S2.

### Analysis

Costs were reported for each country and strata (PSP/MSA) separately. Tests for significant differences between MSA and PSP were not conducted at this stage, as there could be differences between the samples in terms of patient characteristics that might also influence costs. The existence of significant differences in costs was, rather, explored using multivariate analyses. Two regression models were produced. In the first model the dependent variable was the total service costs excluding unpaid care costs. The second model used unpaid care costs as the dependent variable. Variables for inclusion in these two models were chosen because they described features that pre-disposed clients to use services (age, gender), features that enabled them to access services (educational attainment) and features that described their illness (PSP or MSA, disease duration, cognition, symptom severity) [26]. Cognition was measured with the MMSE (dichotomised into two groups with scores 0–27 and 28–30 due to the data distribution) whilst the NNIPPS-PPS total score was used as a global index of disease severity. Given the different service systems in each country and the fact that the unit costs (for reasons described above) may not be directly comparable, we have conducted separate regression analyses for each country and controlled for clustering in study sites.

Cost data are typically positively skewed and this can lead to similarly skewed residuals representing a violation of one of the assumptions on which ordinary least squares models are based. One option for addressing this is to use bootstrapping methods which make no assumption about the underlying data distribution. However, the need for modelling the actual data distribution has been advocated [27]. We have here used a general linear model and specified that the data follow a gamma distribution (which cost data frequently do) and also specified a log link function. This allowed the proportional impact of a unit change in the independent variables on cost to be assessed which was more appropriate than assessing the direct impact given that we were analysing data from quite different service systems.

## Results

Of the 760 patients in the ITT cohort usable data on service use was available at baseline for 742 participants (PSP n = 352, MSA n = 390). Population characteristics are shown in Table 1. The mean age was similar for all countries, and in each the PSP patients tended to be slightly older. In the UK there were relatively fewer women in the sample compared to France and Germany. Clinical characteristics (disease duration and severity) were very similar for PSP and MSA patients and in each country.

**Table 1:** Patient characteristics.

	France		Germany		UK	
	PSP (n = 149)	MSA (n = 158)	PSP (n = 98)	MSA (n = 134)	PSP (n = 105)	MSA (n = 98)
Age (years), mean (SD)	69.3 (7.3)	63.2 (8.3)	67.3 (5.6)	61.3 (7.5)	66.1 (6.7)	62.0 (9.2)
Female, n (%)	75 (48)	79 (48)	39 (39)	67 (50)	40 (38)	34 (34)
Education, n (%)						
*Illiterate*	3 (2)	4 (3)	0 (0)	0 (0)	0 (0)	0 (0)
*Primary school*	52 (34)	47 (29)	37 (37)	40 (30)	18 (17)	15 (15)
*Secondary school*	66 (43)	59 (36)	48 (48)	64 (47)	63 (60)	51 (52)
*Higher schooling*	17 (11)	19 (12)	5 (5)	9 (7)	13 (12)	16 (16)
*University/College*	17 (11)	34 (21))	10 (10)	22 (16)	11 (11)	16 (16)
Clinical Global Impression score, mean (SD)	3.8 (1.0)	3.8 (0.9)	3.7 (1.0)	3.5 (0.9)	3.4 (0.9)	3.4 (1.0)
Short Motor Disability Scale, mean (SD)	6.5 (3.6)	6.4 (3.7)	6.5 (3.9)	5.8(3.8)	6.6 (3.6)	5.9 (3.7)
Disease duration (years), mean (SD)	3.9 (1.7)	4.4 (1.9)	3.9 (1.9)	4.3 (1.8)	4.4 (2.2)	4.8 (2.0)
Hoehn andYahr staging scale, mean (SD)	3.6 (0.9)	3.5 (1.0)	3.7 (1.0)	3.4 (1.1)	3.5 (0.9)	3.4 (1.0)
Parkinson's Plus Symptom scale, mean (SD)	91.9 (31.3)	87.8 (30.8)	90.3 (29.4)	80.6 (31.0)	92.4 (30.1)	83.4 (30.8)
Mini Mental State Examination, mean (SD)	24.0 (5.4)	26.8 (2.8)	25.7 (4.2)	28.8 (1.9)	25.7 (3.9)	28.0 (2.3)

There were some similarities across the three countries in the use of services by patients with PSP (Table 2). The majority of patients had contacts with neurologists and around one-third with other specialists during the six-month period, and most had contacts with general practitioners. Most patients had also had adaptations to their homes, had prostheses such as wheelchairs or walking frames and received unpaid care from family or friends. Almost all received medication. Around one-quarter of patients spent some time in a residential or nursing home. There were some country differences to note. Physiotherapists were seen by around two-thirds of patients in France but by under half in the UK. Neurology inpatient care was used by relatively more patients in Germany (although other inpatient care was used by similar proportions). Social worker and speech therapist contacts were more likely in the UK. Nurses were seen by around two-thirds of all patients in France and the UK and fewer in Germany. Patients in Germany were more likely than those elsewhere to have received blood tests, EEGs and MRIs. Patients in France were the least likely to have received unpaid care from family/friends (although two-thirds still did so). For those receiving specific services there were also large differences in the number of contacts. Residential care days were greater in number in Germany and least in the UK. Contacts with physiotherapists, social workers, nurses and speech therapists were far greater in both France and Germany compared to the UK. Of those patients using day care and home helps, the number of contacts was greatest in the UK. Patients who were admitted to hospital In France had shorter lengths of stay than in Germany or the UK. The final row of Table 2 shows that the mean six-month service costs were similar in France and the UK and around one-fifth higher in Germany.

**Table 2:** Service use and costs (2007/8 €s) for patients with progressive supranuclear palsy (PSP).

	France						Germany						UK					
	Users		Contacts^1^		Cost^2^		Users		Contacts^1^		Cost^2^		Users		Contacts^1^		Cost^2^	
	N	%	Mean	SD	Mean	SD	N	%	Mean	SD	Mean	SD	N	%	Mean	SD	Mean	SD
Neurologist	113	77	2.8	2.8	83	117	74	76	4.6	7.3	122	304	85	81	1.8	1.2	425	348
Other doctor	43	29	2.4	2.5	27	68	32	33	1.8	1.1	17	34	39	37	2.4	2.5	123	263
Day patient	24	16	5.7	4.8	278	878	9	9	11.8	13.2	381	1829	11	11	30.8	34.7	936	4157
Residential care	15	10	88.7	79.9	1278	5329	6	6	159.2	42.4	1645	6781	6	6	45.7	60.3	367	2386
Neurology inpat^3^	23	15	7.5	11.0	446	1987	49	50	14.9	10.1	2306	3267	14	13	13.3	15.8	827	3361
Other inpat^3^	23	15	8.3	10.5	785	3404	16	16	19.1	20.3	1834	6407	20	19	18.2	27.3	1715	6743
GP	135	92	4.8	4.0	113	103	92	94	4.9	4.7	88	101	90	86	3.5	2.8	152	176
Physiotherapist	92	63	55.4	31.4	708	789	58	59	27.5	17.8	467	567	45	43	7.5	10.2	153	389
Social worker	10	7	41.8	66.2	1026	8316	1	1	26.0	-	35	347	30	29	2.8	3.4	91	309
Nurse	44	30	94.0	107.6	314	874	14	14	93.1	143.0	434	2047	35	33	9.9	30.2	65	380
Speech therapist	51	35	31.1	20.3	228	439	21	21	19.4	12.1	145	364	44	42	2.4	2.4	37	71
Home help	47	32	85.0	65.2	1859	5985	11	11	46.6	52.5	158	733	25	24	111.0	114.3	1009	5405
Blood test	73	49	1.7	1.6	21	37	78	79	2.7	3.0	65	88	45	43	2.4	4.2	26	74
CT scan	16	11	1.0	0.0	18	54	16	16	1.1	0.3	19	44	8	8	1.0	0.0	17	60
EEG	17	11	1.0	0.0	5	14	33	33	1.3	0.8	11	20	7	7	1.0	0.0	3	10
MRI	30	20	1.2	0.4	79	173	45	46	1.1	0.4	111	138	29	28	1.1	0.3	133	225
Prostheses	84	54	-	-	31	37	52	52	-	-	35	42	47	45	-	-	29	38
Adaptations	65	42	-	-	74	132	48	48	-	-	107	143	47	45	-	-	82	122
Unpaid care^4^	99	66	61.6	64.2	15814	24077	79	81	57.1	51.7	21574	25183	92	88	50.2	48.9	19327	21770
Medication	144	92	-	-	195	205	88	87	-	-	196	306	89	85	-	-	130	207
Total	149	100	-	-	24491	25231	98	100	-	-	30643	26209	105	100	-	-	25655	22823

1 Data on contacts are only for those using services,

2 Data on costs are for whole sample,

3 Contacts refer to number of days,

4 Contacts refer to weekly hours.

There were very similar patterns of service use for patients with MSA (Table 3). Again, most patients had contacts with neurologists and general practitioners, and many had contacts with other specialists. Residential care was used less in Germany. Most patients received medication and had help from family and friends. Once again, there were fewer contacts with physiotherapists, nurses and speech therapists in the UK compared to France and Germany. The UK patients again had a higher number of day care contacts than the other countries. Mean six-month costs were highest in France, followed closely by Germany. Total costs in the UK were one-third lower in the UK compared to France.

**Table 3:** Service use and costs (2007/8 €s) for patients with multiple system atrophy (MSA).

	France						Germany						UK					
	Users		Contacts^1^		Cost^2^		Users		Contacts^1^		Cost^2^		Users		Contacts^1^		Cost^2^	
	N	%	Mean	SD	Mean	SD	N	%	Mean	SD	Mean	SD	N	%	Mean	SD	Mean	SD
Neurologist	137	87	2.8	1.6	99	116	111	83	4.6	4.4	130	169	86	87	1.6	1.2	434	400
Other doctor	49	31	2.2	2.0	27	61	67	50	3.1	2.9	52	98	33	33	2.2	1.4	103	184
Day patient	37	23	4.8	3.8	342	850	4	3	4.8	2.9	51	332	8	8	32.4	42.8	759	4200
Residential care	11	7	94.9	66.9	954	4349	1	1	174.0	-	224	2605	7	7	84.4	89.9	839	4373
Neurology inpat^3^	19	12	8.5	7.7	395	1504	51	38	16.5	11.0	1982	3327	15	15	7.7	4.8	547	1554
Other inpat^3^	28	18	15.5	33.6	1664	8481	21	16	16.7	16.2	1445	4925	17	17	8.0	8.5	676	2241
GP	145	92	5.1	4.1	126	114	115	86	5.4	7.3	92	161	86	87	3.4	2.9	137	174
Physiotherapist	108	68	54.2	33.2	773	808	104	78	37.8	21.5	864	733	43	43	9.3	11.3	205	490
Social worker	18	11	22.3	53.5	560	4600	4	3	1.5	0.6	3	21	28	28	3.5	3.1	86	207
Nurse	44	28	113.4	128.1	365	1026	11	8	117.3	132.0	321	1626	46	47	11.8	29.3	107	440
Speech therapist	52	33	34.4	24.0	240	500	44	33	21.8	16.2	234	475	37	37	3.4	4.2	59	195
Home help	33	21	88.2	65.2	627	1843	9	7	60.4	52.7	358	2600	13	13	117.1	163.2	347	1219
Blood test	68	43	1.9	2.1	21	43	93	69	2.4	3.1	50	86	44	44	2.0	1.7	22	38
CT scan	11	7	1.0	0.0	13	50	15	11	1.2	0.6	15	46	8	8	1.0	0.0	18	62
EEG	8	5	1.3	0.5	3	13	32	24	1.3	0.8	8	18	6	6	1.2	0.4	3	12
MRI	25	16	1.0	0.2	56	136	41	31	1.1	0.3	72	114	16	16	1.1	0.3	78	183
Prostheses	89	54	-	-	35	42	67	50	-	-	36	45	45	46	-	-	23	31
Adaptations	55	34	-	-	76	133	62	46	-	-	120	177	35	35	-	-	79	144
Unpaid care^4^	110	70	77.4	72.2	21130	28581	103	77	49.8	47.6	19036	24049	75	77	42.2	49.4	14186	20639
Medication	157	96	-	-	362	334	120	89	-	-	317	509	90	91	-	-	226	326
Total	158	100	-	-	28924	29317	134	100	-	-	25645	24284	98	100	-	-	19103	21372

1 Data on contacts are only for those using services,

2 Data on costs are for whole sample,

3 Contacts refer to number of days,

4 Contacts refer to weekly hours.

The distribution of service costs (excluding unpaid care from family/friends) shows that in France, social care accounts for around one-third of PSP costs whilst in Germany and the UK the main contributor to PSP costs is inpatient care (Table 4). For MSA patients, inpatient care is the main contributor to cost in each country. Medication accounts for a very small amount of cost. Unpaid care from family/friends accounts for most of the costs. For PSP the contribution is 68% in France, 73% in Germany and 76% in the UK. For MSA the figures are 76%, 75% and 75% respectively.

**Table 4:** Distribution of service costs (%).

	PSP			MSA		
Service	France	Germany	UK	France	Germany	UK
Hospital doctor	1.5	1.6	8.7	1.9	2.8	11.3
Day patient	3.7	4.7	14.8	5.1	0.8	16.1
Residential care	16.9	20.2	5.8	14.2	3.5	17.8
Inpatient	16.2	50.7	40.2	30.6	53.4	25.8
GP	1.5	1.1	2.4	1.9	1.4	2.9
Other health professional	16.5	12.6	4.0	20.5	22.1	7.9
Social care	38.1	2.4	17.4	17.6	5.6	8.9
Investigations/tests	1.6	2.5	2.8	1.4	2.3	2.6
Prostheses/adaptations	1.6	2.2	1.9	1.9	3.1	2.2
Medication	2.5	2.2	2.1	5.1	4.9	4.7

**Figure 1:**
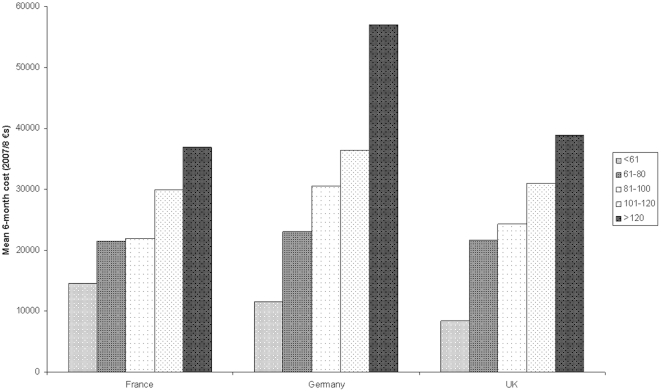
Total costs by symptom severity based on PPS scale (PSP patients).

**Figure 2:**
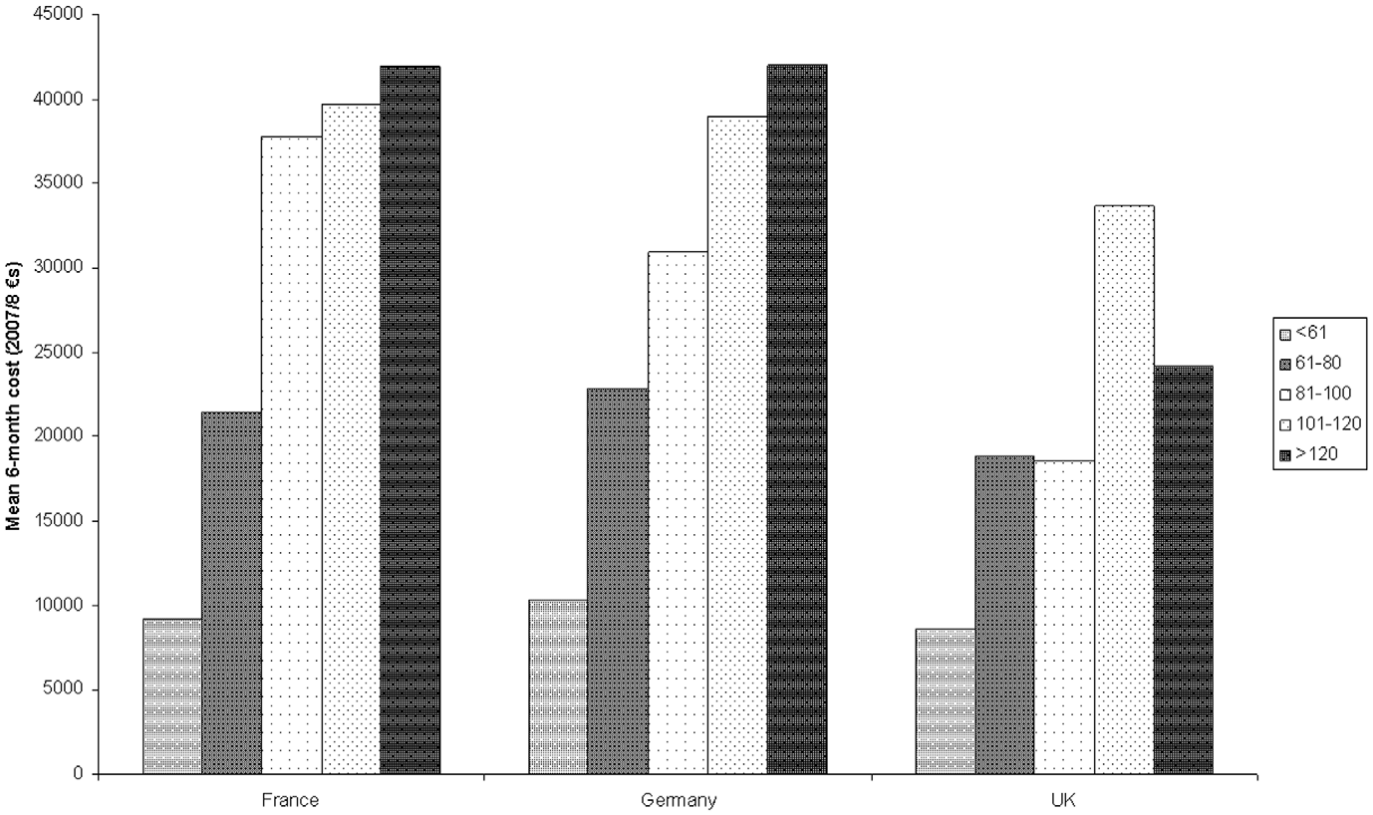
Total costs by symptom severity based on PPS scale (PSP patients).

Figures 1 and 2 show the relationship between total mean cost and symptom severity as measured with the NNIPPS-PPS scale. It can be seen that costs increase with severity in each country for PSP patients. This is also generally the case for MSA patients, although in the UK the patients with the highest severity do not have the highest costs.

The regression analyses for formal care costs (i.e. excluding unpaid care from family/friends) reveals that costs were significantly associated with the NNIPPS-PPS scale, and this relationship was very similar in each country. In France, a one-unit increase in the PPS was associated with a 1.9% increase in costs, whilst in Germany and the UK the figure was 1.7%. In each country there was also an inverse relationship between formal care and unpaid care costs. If the latter increase by €1000 then there is a reduction in formal care costs of 1.2% in France and 1.1% in both Germany and the UK. In France women had formal care costs that were 35% higher on average than for men and those with further education had 63% higher costs than those with the most limited education. In Germany participants with MMSE scores between 28 and 30 had costs that were 48% higher than those with lower scores.

Unpaid care costs were also significantly associated with NNIPPS-PPS scores, with a slightly greater impact of a one-unit increase in Germany (2.2%) compared to France (1.6%) and the UK (1.3%). The inverse relationship with formal care costs was again apparent with similar impacts one a €1000 increase in France (3.7%), Germany (4.1%) and the UK (3.5%). Secondary education was associated with significantly lower unpaid care costs in both France and the UK, with the latter also having significantly lower costs for patients with a university education. In France, patients with PSP had significantly lower unpaid care costs than those with MSA whilst in the UK women had significantly lower costs than men.

There was no relation between costs, formal or unpaid, and age or disease duration.

## Discussion

This is the first study of the costs of providing care for people with PSP and MSA, and we have found that patients with PSP and MSA use a wide range of services and that the mean six-month costs reported are substantial. Most of the costs are related to unpaid care. The costs detailed here are for only three countries and any extrapolation needs to be made with caution. However, using the lowest and highest costs for PSP (€24491 and €30643) and for MSA (€19103 and €28924) and the prevalence figures reported in the introduction suggests annual costs in the 27-country European Union of €1.2–2.7 billion for PSP and €1.1–3.0 billion for MSA. These are likely to be underestimates as the data used in these analyses relate to the six months prior to inclusion in the study (although average disease duration at that point was still approximately 4 years).

Service and unpaid family costs were higher for patients with higher scores on the NNIPPS-PPS which is what we would expect if resources are more likely to be required by those with greater levels of symptom severity. This suggests that interventions which successfully modify disease progression may reduce the economic burden of these conditions. Data on changes in the NNIPPS-PPS have been reported elsewhere [21]. If an intervention was able to reduce the rate of progression by 30% this would lead to NNIPPS-PPS scores being 7.5 points lower for PSP and 5.4 points lower for MSA. While costs will still increase with progression, the regression models in Tables 5 and 6 suggest that services will be 13–14% lower for PSP and 9–10% lower for MSA. For unpaid care costs the reductions will be 10–17% and 7–12% lower respectively.

**Table 5:** Regression analysis of formal service costs.

	France			Germany			UK		
	B	95% CI LL	95% CI UL	B	95% CI LL	95% CI UL	B	95% CI LL	95% CI UL
PSP strata^1^	−0.034	−0.39	0.32	0.21	−0.11	0.53	0.053	−0.34	0.45
Age	0.007	−0.0094	0.023	0.012	−0.011	0.034	−0.0035	−0.032	0.025
Female gender^2^	**0.35**	**0.093**	**0.61**	0.28	−0.076	0.63	−0.057	−0.34	0.22
Duration of disease	−0.0038	−0.076	0.069	−0.056	−0.12	0.011	−0.0033	−0.1	0.096
Secondary education^3^	0.27	−0.049	0.6	0.036	−0.37	0.44	−0.12	−0.51	0.28
Further education^3^	**0.63**	**0.24**	**1**	−0.13	−0.59	0.34	0.36	−0.12	0.85
University education^3^	0.23	−0.19	0.64	−0.03	−0.35	0.29	−0.16	−0.84	0.51
PPS score	**0.019**	**0.013**	**0.025**	**0.017**	**0.012**	**0.022**	**0.017**	**0.012**	**0.023**
MMSE 28–30^4^	0.13	−0.13	0.39	**0.48**	**0.099**	**0.87**	−0.26	−0.66	0.14
Unpaid care costs^5^	**−0.012**	**−0.015**	**−0.0092**	**−0.011**	**−0.018**	**−0.0047**	**−0.011**	**−0.018**	**−0.046**

1 compared to MSA strata,

2 compared to males,

3 compared to illiterate or primary education only,

4 compared to MMSE score 0–27,

5 in €000s.

**Table 6:** Regression analysis of unpaid care costs.

	France			Germany			UK		
	B	95% CI LL	95% CI UL	B	95% CI LL	95% CI UL	B	95% CI LL	95% CI UL
PSP strata^1^	**−0.34**	**−0.68**	**−0.0057**	0.058	−0.19	0.31	0.12	−0.3	0.54
Age	0.015	−0.013	0.042	0.015	−0.003	0.032	0.0089	−0.016	0.033
Female gender^2^	−0.25	−0.61	0.11	0.063	−0.081	0.21	**−0.4**	**−0.6**	**−0.2**
Duration of disease	−0.011	−0.12	0.1	0.0066	−0.048	0.061	0.0064	−0.047	0.06
Secondary education^3^	**−0.34**	**−0.68**	**−0.0038**	0.11	−0.32	0.54	**−0.48**	**−0.7**	**−0.26**
Further education^3^	−0.68	−1.51	0.14	0.051	−0.52	0.62	−0.41	−0.95	0.13
University education^3^	−0.37	−0.8	0.06	−0.11	−0.53	0.3	**−1.2**	**−1.8**	**−0.62**
PPS score	**0.016**	**0.0063**	**0.026**	**0.022**	**0.016**	**0.028**	**0.013**	**0.0063**	**0.02**
MMSE 28–30^4^	0.045	−0.37	0.46	0.19	−0.15	0.52	−0.28	−0.64	0.083
Formal care costs^5^	**−0.037**	**−0.056**	**−0.018**	**−0.041**	**−0.072**	**−0.009**	**−0.035**	**−0.047**	**−0.024**

1 compared to MSA strata,

2 compared to males,

3 compared to illiterate or primary education only,

4 compared to MMSE score 0–27,

5 in €000s.

The costs of unpaid care were significantly lower for women then men in the UK. Life expectancy and caring roles help to explain this finding, although it is surprising that it was not observed in France or Germany. There was an inverse relationship between unpaid family care costs and service care costs, indicating that one substitutes for the other (although the percentage change associated with a €1000 increase is not great. Other variables were associated with cost but not in a consistent way.

Based on studies using a similar methodology, the six-month service costs of multiple sclerosis are around £8500 in the UK and €10000 in Germany [28], [29]. Likewise, the six-month costs of Alzheimer's disease in the UK is approximately €13000 per person and schizophrenia €5500 [30], [31]. Whilst these figures indicate the high care needs of PSP and MSA relative to other conditions, it should be recognised that the total ‘burden’ will be less given the lower prevalence rates. Of particular interest, and in common with other degenerative conditions, is the fact that unpaid family care accounts for most of the cost [32].

There were a number of limitations with the study presented here. First, in order to measure service use comprehensively it was necessary to rely on patient self-report, with help from a carer if necessary. Whilst the schedule used to measure service use is well developed and has been used in numerous other studies, it may still be the case that for some patients recall was difficult and this would have led to inaccuracies. A number of studies have suggested that patient recall of service use can be acceptable [33]–[35]. Another study noted a difference between self-report and administrative records, but pointed out that it was unclear which was more accurate [36]. Mirandola et al indicate that agreement between *total* costs based on self-report and administrative systems is good, but that special care is required when focussing on individual cost components [37]. Second, there were within- and between-country variations in the way in which unit costs were calculated. Between-country variations were addressed to some extent by including country variables in the regression models (and these were not statistically significant). Data for France and Germany consisted of a mixture of actual costs and charges. It is unclear to what extent the inclusion of the (second-best) charge data will have had on the results. If charges were approximately equal to costs then any effects would be small. Third, this paper only examines costs and not the quality of care provided. It may be that some services are not actually having a beneficial impact on health or quality of life (in which case the costs would be too high) or there may be unmet needs (in which case the costs may be too low). Finally, the regression models attempted to identify predictors of cost, but clearly some patient characteristics that could have influenced cost would have been unmeasured.

In conclusion, these results show PSP and MSA to have substantial economic costs, which reveals the many care inputs that these patients require. Costs are appropriately related to indicators of illness severity. Of great importance is the input provided by unpaid carers (family and friends). Costs are significantly associated with symptom severity and if this can be modified then substantial reductions may be realised.

## Supporting Information

Appendix S1 Client Service Receipt Inventory.
(DOC)

Appendix S2 Unit costs used in cost calculations (Euro's).
(DOC)
