# Supplementary material for: The Economic Costs of Progressive Supranuclear Palsy and Multiple System Atrophy in France, Germany and the United Kingdom
Source: PLoS One. 2011 Sep 8;6(9):e24369. doi: 10.1371/journal.pone.0024369 (PMC3169589; doi:10.1371/journal.pone.0024369)
Supplement: Appendix S1 — Client Service Receipt Inventory. (DOC) [file pone.0024369.s001.doc]

**Appendix S1: Client Service Receipt Inventory**

CLIENT SERVICE RECEIPT INVENTORY (1/4)

| 1. Please list any use of the following **hospital and residential services** over the last 6 months | | | |
| --- | --- | --- | --- |
| Service | Have you had contact? No Yes  **0 1** | Unit of  measurement | Number of units received in the last 6 months  (possible range = 1-180) |
| 1-Neurology outpatient visit | ** ** | Attendance |  |
| 2-Other hospital outpatient visit | ** ** | Attendance |  |
| 3-Day hospital (neurology dept) | ** ** | Attendance |  |
| 4-Nursing or residential home | ** ** | Residential days |  |
| 5-Neurology inpatient ward | ** ** | Inpatient days |  |
| 6-Cardiology inpatient ward | ** ** | Inpatient days |  |
| 7-Urology inpatient ward | ** ** | Inpatient days |  |
| 8-Intensive care unit | ** ** | Inpatient days |  |
| 9-Other inpatient ward  (specify) _____________________ | ** ** | Inpatient days |  |

| 2. Have any of these **primary and community care services** been used over the last 6 months? | | | | | |
| --- | --- | --- | --- | --- | --- |
| **Service** | Have you had contact? | Usual location | | **Total no. of contacts** | **Average duration (minutes)** |
|  | No Yes  0 1 | **Care practice**  **1** | **Home**  **2** |  |  |
| 1-General practitioner (GP) | ** ** |  |  |  |  |
| 2-Neurologist | ** ** |  |  |  |  |
| 3-Other doctor *(e.g. cardiologist)* | ** ** |  |  |  |  |
| 4-Physiotherapist | ** ** |  |  |  |  |
| 5-Social worker | ** ** |  |  |  |  |
| 6-Nurse | ** ** |  |  |  |  |
| 7-Speech therapist | ** ** |  |  |  |  |
| 8-Home help | ** ** |  |  |  |  |
| 9-Other service  (specify) _________________ | ** ** |  |  |  |  |

CLIENT SERVICE RECEIPT INVENTORY (2/4)

| 3. Please list any **investigations / diagnostic tests** over the last 6 months |
| --- |

| Service | Have you had this test? No Yes  **0 1** | Total number of investigations in the last 6 months | **Description (if necessary)** |
| --- | --- | --- | --- |
| 1-Magnetic Resonance Image (MRI) | ** ** |  |  |
| 2-CT / CAT scan | ** ** |  |  |
| 3-Electroencephalogram (EEG) | ** ** |  |  |
| 4-Blood test | ** ** |  |  |
| 5-Other investigations / tests | ** ** |  |  |

| 4. Have any of the following **aids or devices** been supplied over the last 6 months? | | |
| --- | --- | --- |
| **Type of aid / device** | **Yes**  **1** | **No**  **0** |
| 1-Wheelchair |  |  |
| 2-Crutches/sticks |  |  |
| 3-Stroller/zimmer frame |  |  |
| 4-Other (please specify)_________________________ |  |  |

| 5. Have there been any **adaptations to the home** because of the patient’s illness? | | |
| --- | --- | --- |
| **Type of adaptation** | **Yes**  **1** | **No**  **0** |
| 1-Stairlift |  |  |
| 2-Shower/bath relocation |  |  |
| 3-Toilet relocation |  |  |
| 4-Redesign kitchen |  |  |
| 5-Medicalised bed |  |  |
| 6-Concrete ramp |  |  |
| 7-Other (e.g. move home)  (please specify)_______________________________________ |  |  |

CLIENT SERVICE RECEIPT INVENTORY (3/4)

| 6. Over the last 6 months, how many hours **informal** **care** per week (on average) has the patient received – because  of their disability or other illness – from *friends and relatives* on the following tasks? | | | | |
| --- | --- | --- | --- | --- |
| **Type of help** | **Have you had help?**  **No Yes**  **0 1** | **Relationship of carer to the patient** | **Average number of hours care per week** | |
| 1-Personal care (e.g. bathing, dressing) | ** ** |  |  | |
| 2-Help inside the home (e.g. cooking, cleaning) | ** ** |  |  | |
| 3-Help outside the home (e.g. shopping) | ** ** |  |  | |
| 4-Other (specify) | ** ** |  |  | |
| What is the principal reason for extra care? | | MSA |  | 1 |
|  |  | PSP |  | 2 |
|  |  | Other illness |  | 3 |

| 7. Have any friends and relatives stayed off work to assist with  the patient’s care because of MSA? | Yes | |  | 1 |
| --- | --- | --- | --- | --- |
|  | No | |  | 0 |
| ***If yes***: For how long have they stayed off work? | Total weeks | | I__I__I__I | |
| Please estimate average income *lost* per week | Currency | | Amount | |
|  | UK-£  FF  DM |      | ____________________ | |

| 8. Over the last 6 months, what **journeys** have been made in order to receive care for your health condition? | | | | | |
| --- | --- | --- | --- | --- | --- |
| **Type of transport** | **Transport used?**  **No Yes**  **0 1** | **Number of journeys** | **Number of travellers** | **Average distance of each return trip (km)** | **Average cost of each journey per person** |
| 1-Private transport (car) | ** ** |  |  |  |  |
| 2-Public transport (train, bus, etc.) | ** ** |  |  |  |  |
| 3-Hospital transport (taxi/car) | ** ** |  |  |  | (leave blank) |
| 4-Hospital transport (ambulance) | ** ** |  |  |  | (leave blank) |

CLIENT SERVICE RECEIPT INVENTORY (4/4)

9.What best describes your current occupation?

|  | Employed full-time | | | |  | | 1 |
| --- | --- | --- | --- | --- | --- | --- | --- |
|  | Employed part-time | | | |  | | 2 |
|  | Unemployed – but available to work | | | |  | | 3 |
|  | Pensioned – through ill-health | | | |  | | 4 |
|  | Retired - through age / choice | | | |  | | 5 |
|  | Housewife/husband | | | |  | | 6 |
|  | Student | | | |  | | 7 |
| If employed: |  | | | |  | |  |
| What gross wage does she/he earn?  (Note 1: gross income = before tax and other deductions)  (Note 2: tick only one time unit and currency) | Time unit | | Currency | | Amount | | |
|  | /Week  /Month  /Year |      | UK-£  FF  DM |      | ___________ | | |
| Have you had to stop or reduce work due to your state of ill-health? | Yes | | | |  | 1 | |
|  | No | | | |  | 0 | |
| *If yes*: How many days in the last 3 months? | Days | | | | I__I__I__I | | |
| or: *How many hours per week less?* | Hours | | | | I__I__I__I | | |
| *If unemployed / retired:* |  | | | |  | | |
| *How long have you been unemployed / retired?* | Years/months | | | | I__I__I / I__I__I | | |
| If currently not working or no longer working: |  | | | |  | |  |
| What is the reason for not / no longer working? | MSA | | | |  | | 1 |
|  | PSP | | | |  | | 2 |
|  | Other illness | | | |  | | 3 |
|  | Not illness-related | | | |  | | 4 |
