# Supplementary material for: The Economic Costs of Progressive Supranuclear Palsy and Multiple System Atrophy in France, Germany and the United Kingdom
Source: PLoS One. 2011 Sep 8;6(9):e24369. doi: 10.1371/journal.pone.0024369 (PMC3169589; doi:10.1371/journal.pone.0024369)
Supplement: Appendix S2 — Unit costs used in cost calculations (Euro's). (DOC) [file pone.0024369.s002.doc]

**Appendix S2. Unit costs used in cost calculations (Euro’s).**

| **Service** | **Unit** | **France** | **Germany** | **UK** |
| --- | --- | --- | --- | --- |
| Neurologist outpatient | Visit | 40.51 | 34.30 | 305.77 |
| Other outpatient | Visit | 40.51 | 34.30 | 140.71 |
| Neurology day patient | Visit | 316.58 | 363.08 | 290.00 |
| Residential care | Day | 149.88 | 173.93 | 140.59 |
| Neurology inpatient | Day | 402.47 | 318.56 | 466.74 |
| Cardiology inpatient | Day | 533.78 | 486.48 | 789.34 |
| Urology inpatient | Day | 692.43 | 486.48 | 468.46 |
| Intensive care unit | Day | 1824.00 | 1400.33 | 2113.63 |
| Other inpatient | Day | 524.36 | 608.51 | 741.86 |
| General practitioner at clinic | Contact  Minute | 35.43  - | 17.75  - | -  3.19 |
| General practitioner at home | Contact  Minute | 23.62  - | 23.57  - | -  5.00+28.40 travel |
| Neurologist (non-outpatient) | Minute | 1.17 | 1.74 | 10.47 |
| Other doctor (non-outpatient) | Minute | 1.53 | 1.74 | 4.69 |
| Physiotherapist | Contact | 21.68 | 29.63 | - |
| Physiotherapist at clinic | Minute | - | - | 0.95 |
| Physiotherapist at home | Minute | - | - | 1.23 |
| Social worker | Minute | 1.48 | 1.93 | 2.59 |
| Nurse | Contact  Minute | -  0.34 | 33.63  - | -  - |
| Nurse at clinic | Minute | - | - | 1.11 |
| Nurse at home | Minute | - | - | 1.56+1.95 travel |
| Speech therapist | Minute | 0.65 | 0.86 | - |
| Speech therapist at clinic | Minute | - | - | 0.89 |
| Speech therapist at home | Minute | - | - | 1.14+3.74 travel |
| Home help | Minute | 0.27 | 0.33 | 0.29 |
| Informal care | Hour | 15.94 | 20.05 | 17.46 |
| MRI | Scan | 354.30 | 220.65 | 451.40 |
| CT | Scan | 177.15 | 110.33 | 225.70 |
| EEG | Scan | 45.49 | 26.86 | 41.77 |
| Blood test | Test | 17.72 | 20.56 | 25.06 |
| Wheelchair ^a^ | Aid | 66.80 | 77.52 | 62.66 |
| Crutches ^a^ | Aid | 26.72 | 31.01 | 25.06 |
| Frame ^a^ | Aid | 35.63 | 41.34 | 33.42 |
| Stair lift ^a^ | Adaptation | 267.19 | 310.07 | 250.63 |
| Shower ^a^ | Adaptation | 163.88 | 190.18 | 153.72 |
| Toilet ^a^ | Adaptation | 139.83 | 162.27 | 131.16 |
| Kitchen redesign ^a^ | Adaptation | 320.63 | 372.09 | 300.75 |
| Medical bed ^a^ | Adaptation | 45.78 | 53.13 | 42.94 |
| Ramp ^a^ | Adaptation | 31.17 | 36.18 | 29.24 |

^a^ Costs are annuitised to a six-month period
